# Efficacy of melatonin with behavioural sleep-wake scheduling for delayed sleep-wake phase disorder: A double-blind, randomised clinical trial

**DOI:** 10.1371/journal.pmed.1002587

**Published:** 2018-06-18

**Authors:** Tracey L. Sletten, Michelle Magee, Jade M. Murray, Christopher J. Gordon, Nicole Lovato, David J. Kennaway, Stella M. Gwini, Delwyn J. Bartlett, Steven W. Lockley, Leon C. Lack, Ronald R. Grunstein, Shantha M. W. Rajaratnam, for the Delayed Sleep on Melatonin (DelSoM) Study Group

**Affiliations:** 1 Monash Institute of Cognitive and Clinical Neurosciences, School of Psychological Sciences, Monash University, Victoria, Australia; 2 Cooperative Research Centre for Alertness, Safety and Productivity, Victoria, Australia; 3 CIRUS, Woolcock Institute of Medical Research, University of Sydney, New South Wales, Australia; 4 Sydney Nursing School, University of Sydney, New South Wales, Australia; 5 School of Psychology, Faculty of Social and Behavioural Sciences, Flinders University, South Australia, Australia; 6 Robinson Research Institute, School of Medicine, Discipline of Obstetrics and Gynaecology, University of Adelaide, Adelaide, South Australia, Australia; 7 Department of Epidemiology and Preventative Medicine, Monash University, Victoria, Australia; 8 University Hospital Geelong, Barwon Health, Geelong, Victoria, Australia; 9 Division of Sleep and Circadian Disorders, Departments of Medicine and Neurology, Brigham and Women’s Hospital, Division of Sleep Medicine, Harvard Medical School, Massachusetts, United States of America; 10 Department of Respiratory and Sleep Medicine, Royal Prince Alfred Hospital, New South Wales, Australia; The George Institute for Global Health, UNSW Sydney, AUSTRALIA

## Abstract

**Background:**

Delayed Sleep-Wake Phase Disorder (DSWPD) is characterised by sleep initiation insomnia when attempting sleep at conventional times and difficulty waking at the required time for daytime commitments. Although there are published therapeutic guidelines for the administration of melatonin for DSWPD, to our knowledge, randomised controlled trials are lacking. This trial tested the efficacy of 0.5 mg melatonin, combined with behavioural sleep-wake scheduling, for improving sleep initiation in clinically diagnosed DSWPD patients with a delayed endogenous melatonin rhythm relative to patient-desired (or -required) bedtime (DBT).

**Methods:**

This randomised, placebo-controlled, double-blind clinical trial was conducted in an Australian outpatient DSWPD population. Following 1-wk baseline, clinically diagnosed DSWPD patients with delayed melatonin rhythm relative to DBT (salivary dim light melatonin onset [DLMO] after or within 30 min before DBT) were randomised to 4-wk treatment with 0.5 mg fast-release melatonin or placebo 1 h before DBT for at least 5 consecutive nights per week. All patients received behavioural sleep-wake scheduling, consisting of bedtime scheduled at DBT. The primary outcome was actigraphic sleep onset time. Secondary outcomes were sleep efficiency in the first third of time in bed (SE T1) on treatment nights, subjective sleep-related daytime impairment (Patient Reported Outcomes Measurement Information System [PROMIS]), PROMIS sleep disturbance, measures of daytime sleepiness, clinician-rated change in illness severity, and DLMO time.

**Findings:**

Between September 13, 2012 and September 1, 2014, 307 participants were registered; 116 were randomised to treatment (intention-to-treat *n =* 116; *n =* 62 males; mean age, 29.0 y). Relative to baseline and compared to placebo, sleep onset occurred 34 min earlier (95% confidence interval [CI] −60 to −8) in the melatonin group. SE T1 increased; PROMIS sleep-related impairment, PROMIS sleep disturbance, insomnia severity, and functional disability decreased; and a greater proportion of patients showed more than minimal clinician-rated improvement following melatonin treatment (52.8%) compared to placebo (24.0%) (*P* < 0.05). The groups did not differ in the number of nights treatment was taken per protocol. Post-treatment DLMO assessed in a subset of patients (*n =* 43) was not significantly different between groups. Adverse events included light-headedness, daytime sleepiness, and decreased libido, although rates were similar between treatment groups. The clinical benefits or safety of melatonin with long-term treatment were not assessed, and it remains unknown whether the same treatment regime would benefit patients experiencing DSWPD sleep symptomology without a delay in the endogenous melatonin rhythm.

**Conclusions:**

In this study, melatonin treatment 1 h prior to DBT combined with behavioural sleep-wake scheduling was efficacious for improving objective and subjective measures of sleep disturbances and sleep-related impairments in DSWPD patients with delayed circadian phase relative to DBT. Improvements were achieved largely through the sleep-promoting effects of melatonin, combined with behavioural sleep-wake scheduling.

**Trial registration:**

This trial was registered with the Australian New Zealand Clinical Trials Registry, ACTRN12612000425897.

## Abstract 2

In this randomized trial, Tracey L Sletten demonstrate sleep-promoting effects of melatonin combined with behavioral sleep-wake scheduling in participants with Delayed Sleep-Wake Phase Disorder.

## Author summary

**Why was this study done?:**

- Circadian disruption—and Delayed Sleep-Wake Phase Disorder (DSWPD) specifically—is associated with significant morbidity, including depression, poor academic and work performance, and adverse social and economic outcomes.
- Previous studies examining treatments for DSWPD have typically diagnosed the disorder based on sleep symptomology, not circadian timing, which may result in misdiagnosis of sleep initiation insomnia as DSWPD and thus inappropriate treatment.
- Although exogenous melatonin has been widely advocated as a treatment for DSWPD, the therapeutic potential of the sleep-promoting effects of melatonin have not been evaluated rigorously in a randomised controlled trial in well-characterised DSWPD patients.

**What did the researchers do and find?:**

- DSWPD patients (*n =* 116) with a delayed melatonin rhythm relative to patient desired bedtime (DBT) completed a 4-wk trial of melatonin (0.5 mg) or placebo administered 1 h prior to DBT, combined with a behavioural sleep-wake intervention consisting of attempting sleep at the patient DBT for at least 5 nights per week.
- Relative to baseline, sleep onset time was 34 min earlier, sleep efficiency during the first tertile of sleep (SE T1) was greater, and sleep-related functional impairment was lower in the melatonin group compared to placebo.
- Sleep disturbance, insomnia severity, and functional disability decreased, and a greater proportion of patients showed clinician-rated improvement following melatonin treatment (52.8%) compared to placebo (24.0%).
- The observed improvements in sleep were clinically significant according to clinical practice guidelines for the treatment of circadian rhythm sleep disorders and compared to other established therapeutic approaches.

**What do the findings mean?:**

- Melatonin treatment, when combined with behavioural sleep-wake scheduling, is efficacious for improving sleep disturbances and sleep-related impairments in DSWPD patients and potentially in other patients who show abnormal circadian timing relative to sleep.
- Current melatonin treatment protocols for DSWPD vary in patient diagnostic criteria; vary in the dose, formulation, and timing of melatonin; and may result in low compliance due to the requirement for strict adherence every day.
- These findings contribute to the evidence base to improve the diagnosis of DSWPD and the development of a standardised treatment protocol.

## Introduction

Delayed Sleep-Wake Phase Disorder (DSWPD) is characterised by sleep initiation insomnia when attempting sleep at conventional times and difficulty waking at the required time for daytime commitments such as work or school [1,2]. DSWPD is often associated with chronic sleep restriction [3] and adverse academic, occupational, financial, mental health, and social outcomes [4–6].

The intrinsic basis for DSWPD manifests in a delay in endogenous circadian timing [2,3,7]. Current diagnostic criteria [1], however, do not specifically include circadian timing, which may result in misdiagnosis of sleep initiation insomnia as DSWPD [8]. Individuals may also present with delayed sleep-wake behaviour due to differences in homeostatic sleep drive not related to circadian timing and thus still show differences in sleep onset timing relative to those with healthy sleep [9]. Previous trials examining treatments for DSWPD have typically diagnosed the disorder based on sleep symptomology, not circadian timing [10–14].

Although exogenous melatonin has been suggested for clinical treatment of DSWPD [15], standardised evidence-based therapeutic guidelines are lacking. In addition to the circadian phase-shifting effects of melatonin, the hormone has direct, sleep-promoting effects [16]. The therapeutic potential of these sleep-promoting effects has not been evaluated rigorously in a randomised controlled trial in DSWPD patients. Such a treatment approach would be beneficial for this patient group, which may use melatonin to facilitate sleep at an earlier time only as required, for example, on nights before school or work days.

The aim of this study was to test—in a randomised, double-blind, parallel-groups, placebo-controlled outpatient trial—the efficacy of melatonin (0.5 mg) for DSWPD patients with confirmed delay of the endogenous melatonin rhythm relative to desired or required bedtime using a pragmatic, clinically relevant protocol that included behavioural sleep-wake scheduling (sleeping at desired or required bedtime) for all participants. Specifically, we tested the hypothesis that, compared to placebo, administration of melatonin 1 h prior to the patient’s predetermined desired (or required) bedtime for 4 wk would advance the time of sleep onset. As secondary outcomes, we hypothesised that compared to placebo, melatonin treatment would result in (1) increased sleep efficiency in the first third of the sleep episode (SE T1), (2) reduced subjective sleep-related daytime impairments, (3) reduced self-reported sleep disturbances and daytime sleepiness, (4) a greater proportion of change in the clinician-rated change in severity of illness, and (5) an advance in the timing of the endogenous circadian pacemaker.

## Methods

### Study design

The Delayed Sleep on Melatonin (DelSoM) study was a multicentre, double-blind randomised controlled trial conducted at 3 study sites in Australia: Monash University in Melbourne, Woolcock Institute of Medical Research in Sydney, and Flinders University in Adelaide. Data were collected between September 2012 and September 2014. The study protocol was approved by the Monash University Human Research Ethics Committee, the University of Sydney Human Research Ethics Committee, University of Adelaide Human Research Ethics Committee, and Southern Adelaide Clinical Human Research Ethics Committee. The trial was registered with the Australian New Zealand Clinical Trials Registry (ACTRN12612000425897).

### Participants and recruitment

Participants were males and females aged between 16 and 65 y with body mass index between 18 and 35 kg/m^2^ who had daytime commitments on ≥5 d per week. They had no reported history of psychiatric disorders in the past 12 mo (other than depression), reported no illicit drug use for at least 12 mo, were nonsmokers, and consumed low habitual amounts of caffeine (<300 mg/d) and alcohol (<14 units/wk). Participants were not working regular night shifts and had not travelled across more than 2 time zones in the previous 2 mo. Participants were recruited via radio, newspaper, television and poster advertisements, and via clinician referrals. Potential participants were identified as displaying symptoms of DSWPD via online questionnaire and were interviewed via telephone before attending a screening visit. Following consent, patients were assessed by a sleep physician who confirmed they met International Classification of Sleep Disorders-2 diagnostic criteria for DSWPD [17] based on clinical interview. Participants provided written informed consent prior to study commencement.

### Baseline questionnaire, sleep-wake, and circadian phase assessments

DSWPD diagnostic criteria were assessed via clinical interview, and because 7-d collection of actigraphy and sleep diaries were used as assessment outcomes at baseline, they were not used as part of diagnosis. After the clinical interview, a sleep physician completed the Clinical Global Impression-Severity (CGI-S) scale [18] as a measure of global functioning prior to treatment. Participants completed pretreatment surveys, including the Pittsburgh Sleep Quality Index (PSQI) [19] for subjective sleep quality, Epworth Sleepiness Scale (ESS) to assess daytime sleepiness [20], Morningness-Eveningness Questionnaire to assess diurnal preference [21], Insomnia Severity Index (ISI) [22], Beck Depression Inventory version 2 (BDI-II) as a measure of current experience of depressive symptoms [23], and Beck Anxiety Inventory [24]. The Sheehan Disability Scale (SDS) was completed to assess functional impairments in 3 interrelated domains: work/school, social life, and family life, in addition to the rate of absenteeism and presenteeism [25].

For 7 d, participants were monitored during their habitual sleep-wake schedule and regular daytime commitments via sleep diaries and wrist actigraphy (Actiwatch-L, Actiwatch-2, or Actiwatch Spectrum; Philips Respironics, Bend, OR). Participants also documented their daily schedules to confirm daytime commitments on at least 5 consecutive days per week. On the last morning, 1 to 4 h after waking, participants completed the Patient Reported Outcomes Measurement Information System (PROMIS) as measures of sleep disturbance and sleep-related daytime impairments, including symptoms of sleepiness, irritability, and difficulty concentrating [26].

After the outpatient sleep-wake assessment, participants attended the laboratory for assessment of circadian phase via salivary dim light melatonin onset (DLMO). Participants arrived 6 h before their self-reported habitual sleep onset time and remained awake in a light-proof, sound-attenuated, temperature-controlled suite until at least 2 h after their habitual sleep onset time. Ambient light was maintained at <10 lux (measured in the direction of gaze at standard height of seated eye level, 137 cm from the floor), and saliva samples (approximately 2 mL) were collected hourly. Participants remained seated with limited movement for 20 min before each sample and were not permitted to consume food or beverages within 10 min of each sample. They were permitted to watch television (<10 lux), read, and engage in quiet activities between samples. Samples were collected (Salivette, Sarstedt, Numbrecht, Germany) as previously described [27] and stored at −20 °C. After the final sample, participants were transported home.

Saliva (200 μl) was assayed in triplicate for melatonin by radioimmunoassay within 1 wk of collection, using procedures developed at the University of Adelaide [28] and reagents provided by Buhlmann Laboratories (Allschwil, Switzerland). Limit of detection of the assay was 1 pg/mL, the inter-assay coefficient of variations were 7.4% at 4.41 pg/mL and 10.7% at 48.14 pg/mL, and the intra-assay coefficient of variation was 9.9%. The time of DLMO for each participant was calculated as the time that melatonin concentrations crossed and remained above a threshold of 2.3 pg/mL (10 pM), calculated from linear interpolation between the samples immediately before and after the threshold [27]. Participants were classified as having a delayed circadian phase angle if DLMO occurred within 30 min before their reported desired bedtime (DBT) or any time after DBT. DBT was derived from the response to the following question: ‘On the night before school or work, what time would you need to go to bed in order to feel fully rested in the morning?’ It was considered that, for individuals with DLMO before DBT, abnormal timing of the central circadian pacemaker was not likely to be the primary cause of their sleep initiation complaints at DBT. Although these individuals present with similar difficulties in sleep initiation, the phenotype of circadian rhythm sleep disorder is not present [8], and the optimal treatment regimens for each may not be the same. To account for potential measurement error in DLMO and/or DBT, individuals with DLMO occurring up to 30 min before DBT were also classified as having circadian delay. In cases of actigraphy malfunction or noncompliance (Actiwatch worn during <5 sleep episodes or not worn for the 2 nights prior to DLMO assessment), participants repeated the sleep-wake monitoring after the DLMO assessment (*n =* 9).

### Randomisation and treatment

Individuals with a confirmed delayed circadian phase angle were randomised to treatment with placebo or 0.5 mg fast-release melatonin (Pure Encapsulations; Sudbury, MA). Participants were assigned to treatment arms using permuted blocks randomisation (*n =* 6) in a 1:1 ratio. An externally appointed biostatistician was responsible for randomisation procedures. Administration of medication type was double-blinded. Blinding of medication conditions was conducted by a pharmacist at Monash University. Medication was dispensed by a university (Monash) or local (Woolcock, Flinders) pharmacist. Placebo capsules were lactose and cellulose and were identical in size and appearance to the melatonin capsule.

Participants were provided with 28 medication capsules and instructed to take a capsule at the same clock time each night 1 h before their fixed DBT and to attempt sleep at their DBT each night prior to daytime commitments, as the behavioural sleep-wake scheduling component of the protocol, for at least 5 consecutive nights of every 7 nights for 4 consecutive weeks. To assess compliance with medication and inform analyses, participants self-reported the precise time of administration each night and returned unused capsules at study end. During the treatment period, sleep-wake monitoring was continued with sleep diaries and wrist actigraphy. Participants continued to document their daily work schedules, and at the end of each week, they completed the PROMIS. Participants were contacted by researchers via telephone each week to promote compliance with the protocol and monitor potential adverse events.

The protocol for melatonin administration an hour before DBT rather than habitual bedtime was developed by taking into consideration the available evidence of the phase resetting and sleep-promoting effects of melatonin and what was deemed to be achievable in a clinical setting based on our experience. First, we determined that melatonin would be administered on nights in which the participant needed to advance their bedtime rather than requiring adherence every night. We expected melatonin to be efficacious using this approach because of the acute sleep-promoting effects of the hormone and also the immediate phase-shifting effect. A single night of administration of a melatonin agonist can produce a phase advance in humans [29,30], and the rhythm of light-induced c-Fos gene expression in the suprachiasmatic nuclei is immediately shifted following melatonin administration in animal models [31]. The dose of melatonin we used was selected based on previous work showing no difference in improvements in sleep latency or sleep efficiency (SE) between 0.5 mg and 5 mg compared to placebo in healthy volunteers in a controlled setting [32]. We also selected the minimal effective dose to avoid the potential for residual (next-morning) effects on neurocognitive function and on the circadian pacemaker, which may occur if higher doses of melatonin remain in circulation for a longer duration. We do note, however, that the phase-response curves reported for 0.5 mg and 3 mg melatonin do not markedly differ [33]. Whether doses exceeding 3 mg of melatonin alter the phase-resetting response is presently unclear. A standard fast-release preparation of melatonin was administered to rapidly induce high circulating levels close to the time of desired sleep onset but to avoid residual (circadian phase-shifting) effects towards the end of the sleep episode. The metabolism and pharmacokinetics of such melatonin preparations have been well described [34,35]. In one study in which the dose-response effects of melatonin were examined, for 0.5 mg of melatonin, the time taken to reach maximum concentration was 30 min, and the elimination half-life was 42.6 + 12.6 min [36].

### Post-treatment assessments

Within 7 d of treatment end, participants met a sleep physician for completion of post-treatment CGI-S. Participants completed the Patients’ Global Impression of Change (PGI-C) Scale [18] and repeated the PSQI, ESS, ISI, SDS, BDI-II, and BAI as post hoc secondary outcomes.

In a subset of participants at the Monash site (*n =* 49/104; *n =* 27 melatonin; and *n =* 22 placebo), a follow-up DLMO assessment was conducted 24 h after completing treatment. Post-treatment DLMO assessments were conducted in the participants’ home. They remained seated in a dimly lit room (<10 lux at participant eye level, vertical plane) and collected saliva samples every hour from 5 h before until 2 h after their treatment bedtime. During the 7-h saliva collection, participants were able to watch television, read, and engage in quiet activities between samples. Research staff measured the at-home lighting to ensure dim conditions prior to the first sample and remained present for the first sample. Home saliva sampling for DLMO assessment under controlled conditions of light and sample timing correlate highly with laboratory assessments [37]. Post-treatment DLMO assessments were only available at 1 site (Monash) due to researcher availability and participants opted-in to this aspect of the protocol.

### Statistical analysis

The original study sample size was planned based on a meta-analysis of the efficacy of melatonin for DSWPD in adults, which reported a 0.70-h mean difference in sleep onset time between melatonin and placebo [38]. The pooled results in the meta-analysis primarily based on actigraphic data suggested that a sample size of *n =* 79 patients per group (158 in total) would have 90% power at 0.05 significance level to show a 0.94 h (SD = 1.82) difference in sleep onset time between groups. The sample size was not reached due to budgetary constraints and slower than anticipated patient recruitment.

Intention-to-treat analyses were conducted as per the Consolidated Standards of Reporting Trials (S1 CONSORT Checklist). We defined the intention-to-treat population as all randomised individuals for whom trial medication was dispensed (*n =* 116). Actigraphic and subjective sleep variables were calculated for all nights on which medication was taken because DSWPD symptoms manifest when the patient attempts to sleep at their desired or required bedtime (prior to school or work commitments the following day), and not when they sleep at the biologically driven time. Therefore, the trial design required that the medication be taken on nights prior to school or work commitments. In post hoc sensitivity analyses, we included only patients who completed the trial, using only nights during which they took medication within 30 min of the scheduled time and attempted sleep within 1 h of taking the medication (*n =* 104; completed treatment). No missing value imputation or substitution was performed.

Sleep diary reports of bedtime, sleep offset time, sleep onset latency (SOL), and wake after sleep onset (WASO) were used to calculate the subjective sleep variables of time in bed (TIB), sleep onset time, total sleep time (TST), and SE (calculated between sleep onset and sleep offset). Bed and wake times reported in sleep diaries were used to identify sleep episodes for actigraphy analysis in Actiware 5 software (Philips Respironics, Bend, OR). For actigraphic analyses, discrepancies between sleep diaries and actigraphy of ≥60 min required correction to match objective actigraphy. If the reported subjective bedtime was ≥60 min prior to a sustained substantial reduction in activity and light levels, bedtime was adjusted to the time of activity and light reduction. If reported wake time was ≥60 min after a sustained substantial increase in activity and light levels, wake time was adjusted to match the time of increased activity and light. An independent researcher visually reviewed alignment between diary and actigraphic bed and wake times, and discrepancies were resolved by discussion and agreement between 2 researchers (JMM and MM) before unblinding. Actiware software sensitivity was set to medium (40 activity counts) to determine each 1-min epoch as sleep or wake.

The following actigraphic variables were computed for main sleep episodes: TIB, sleep onset time, sleep offset time, rise time, TST (sleep onset to wake time minus awakenings), SOL (minutes taken to fall asleep), SE (proportion of TIB that was sleep), and WASO (minutes of wakefulness after sleep onset). Sleep onset was established as the first epoch in a series of at least 10 min in which no more than 1 epoch contained measured activity. Sleep episodes were divided into tertiles (from actigraphic bedtime to rise time), and SE was calculated separately for each tertile.

Demographic and clinical characteristics were compared between placebo and treatment groups at baseline for intention-to-treat (*n =* 116) and completed treatment (*n =* 104) populations. Categorical baseline data were summarised as frequencies as percentages, and comparisons between treatment groups were conducted using the chi-squared statistic. Continuous or interval data were reported as means with their standard deviations, and linear regression was used for group comparisons. Classifications of CGI and PGI-C were determined post hoc based on the distribution of responses.

To establish whether there were study group differences in changes in outcomes from baseline to intervention period, linear mixed models were used allowing for a random intercept and slope. Linear mixed models compared the 7-d baseline period average to the average of treatment nights for sleep parameters (actigraphic and sleep diary: bedtime, sleep onset and offset times, TIB, TST, SOL, SE, and WASO; actigraphic: rise time and SE tertiles 1, 2, and 3). The linear models used restricted maximum likelihood estimation and variance component structures for residuals, and random effects were modelled by considering the best-fitting structure. Mean change in sleep parameters from baseline nights to treatment nights were compared between treatment groups using an interaction term for group (melatonin versus placebo) and treatment period (baseline versus treatment).

Ordinal linear regression models were used to establish group comparisons of ISI, PSQI, ESS, and SDS absenteeism and presenteeism. Binary outcomes (PSQI, PGI-C, ESS) were analysed using logistic regression, while ordinal logistic regression was used for groupings of ISI and DSPD severity (i.e., CGI-S). Because these data were collected at 2 time points (baseline, end of treatment), robust cluster sandwich estimators were employed to account for repeated measures. Analyses were conducted using Stata Statistical software release 14.1 (StataCorp 2015, College Station, TX). Statistical significance was defined as a *P* value less than 0.05. Unless otherwise stated, data are presented as mean ± SD.

## Results

**Fig 1:**
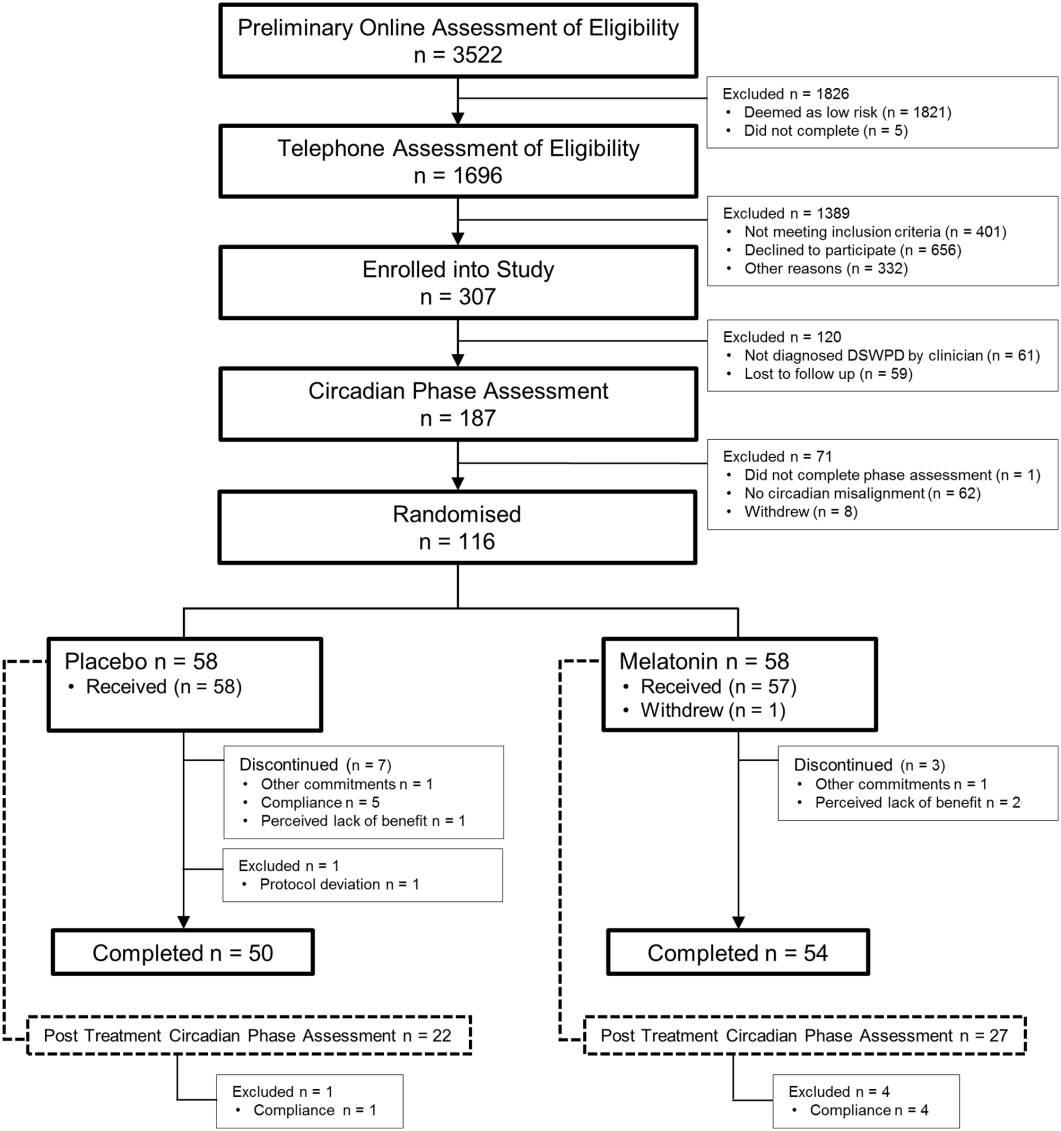
Participant recruitment and study enrolment flow chart.

Preliminary online screening was completed by 3,522 individuals, of whom 307 were enrolled in the study, 187 attended the laboratory-based circadian phase assessment, and 116 were randomised to treatment (Fig 1). Of the 186 who completed the circadian phase assessment, 62 (33.3%) did not have circadian misalignment between DLMO and DBT (defined as DLMO <30 min before or any time after DBT) and were excluded from the trial. Comparison of sleep-wake and mood characteristics of patients with and without circadian misalignment from this trial are presented elsewhere [8].

Patients were initially screened via online questionnaires followed by telephone assessment, and DSWPD symptomology was confirmed by a sleep physician. Circadian phase assessment identified individuals who had delayed DLMO relative to desired or required bedtime, and these individuals were randomly assigned to treatment. The intention-to-treat population included the 116 patients who were randomised. There were 104 patients in the completed treatment analysis.

Demographic and clinical characteristics of participants in each treatment group are presented in Table 1. Participants in the placebo and melatonin treatment groups did not differ in sex, age, body mass index, morningness-eveningness scores, clinician-derived DSWPD severity, baseline DLMO, DBT, phase angle between DLMO and DBT, or phase angle between DLMO and actigraphic habitual bedtime (Table 1; *P* > 0.05).

**Table 1:** Baseline demographic and clinical characteristics and relationship between DLMO and sleep timing among patients randomised to treatment with placebo or melatonin.

		Intention-to-Treat			Completed Treatment		
		Placebo	Melatonin	*P*	Placebo	Melatonin	*P*
No.		58	58		50	54	
Sex (no. [%])		32 (55) M, 26 (45) F	30 (52) M, 28 (48) F	0.710	25 (50) M, 25 (50) F	28 (52) M, 26 (48) F	0.850
Age (y):	Mean ± SD	28.29 ± 9.96	29.72 ± 9.33	0.426	28.88 ± 10.46	29.94 ± 9.63	0.591
	Median (IQR)	25 (21–31)	27 (24–36)	0.170	25 (21–33)	27.5 (23–36)	0.329
	Range	17–60	17–64		17–60	17–64	
Body mass index (kg/m^2^): Mean ± SD		24.68 ± 4.17	25.50 ± 4.43	0.308	24.66 ± 4.34	25.49 ± 4.43	0.340
Morningness-eveningness questionnaire:	Mean ± SD	23.41 ± 4.44	24.57 ± 4.92	0.187	23.90 ± 4.33	24.57 ± 5.09	0.468
	Median (IQR)	22 (20–27)	24 (21–27)	0.291	22.5 (21–27)	24 (21–27)	0.766
Evening (0–22): no. (%)		31 (53.4)	25 (43.1)	0.265	25 (50.0)	25 (46.3)	0.706
Intermediate (23–43): no. (%)		27 (46.6)	33 (56.9)		25 (50.0)	29 (53.7)	
Morning (>44): no. (%)		0 (0.0)	0 (0.0)		0 (0.0)	0 (0.0)	
DSWPD severity, clinician-rated: no. (%)^b^							
Mild		21 (37)	19 (33)	0.890	18 (37)	18 (33)	0.782
Moderate		30 (53)	33 (57)		25 (51)	31 (57)	
Severe		6 (11)	6 (10)		6 (12)	5 (9)	
Baseline DLMO (hh:mm): Mean ± SD		22:53 ± 1:16	22:54 ± 1:27	0.948	22:46 ± 1:12	22:52 ± 1:27	0.675
DBT (hh:mm): Mean ± SD		22:32 ± 0:56	22:23 ± 1:01	0.419	22:27 ± 0:56	22:25 ± 0:59	0.900
DBT-DLMO (h)^c^:	Mean ± SD	−0.35 ± 0.87	−0.51 ± 1.07	0.371	−0.32 ± 0.85	−0.45 ± 1.06	0.480
	Median (IQR)	−0.25 (−0.83 to 0.35)	−0.14 (−1.00 to 0.30)	0.677	−0.21 (−0.83 to 0.35)	−0.07 (−0.97 to 0.32)	0.848
HBT (hh:mm)^a^: Mean ± SD		00:59 ± 1:27	01:11 ± 1:14	0.419	00:50 ± 1:20	01:08 ± 1:15	0.263
HBT-DLMO (h)^a^^,^^c^: Mean ± SD		2.12 ± 0.97	2.31 ± 1.21	0.358	2.11 ± 0.89	2.28 ± 1.21	0.406
Planned treatment time per protocol (hh:mm): Mean ± SD		21:32 ± 0:56	21:23 ± 1:01	0.419	21:27 ± 0:56	21:25 ± 0:59	0.900

Median (IQR) provided for variables with skewed distribution.

^a^HBT and HBT-DLMO for intention-to-treat groups; placebo *n =* 55; melatonin *n =* 56. HBT and HBT-DLMO for completed treatment groups; placebo *n =* 47; melatonin *n =* 52.

^b^DSWPD severity for intention-to-treat placebo *n =* 57; DSWPD severity for completed treatment placebo *n =* 49.

^c^*P* values based on a *t* test of phase angles of the differences between DLMO and DBT or DLMO and HBT.

Abbreviations: DBT, subjective desired bedtime; DLMO, dim light melatonin onset; DSWPD, Delayed Sleep-Wake Phase Disorder; HBT, actigraphic habitual bedtime; IQR, interquartile range; SD, standard deviation.

On average, patients took capsules (melatonin or placebo) on 21.11 ± 3.18 nights during the 4-wk treatment protocol, with an average of 5.30 ± 1.00 nights per 7-d period. Of the total completed sample (*n =* 104), 66.3% took capsules for at least 5 consecutive nights per week on at least 3 of the 4 wk. Notably, only 5.0% opted to take capsules for 7 nights per week on all weeks. The total number of nights for which patients complied with the treatment protocol (capsule taken and bedtime as instructed) did not differ between groups (536 versus 614 nights for melatonin and placebo groups, respectively; *P* > 0.05). The circadian timing of scheduled treatment (timing of capsule relative to baseline DLMO) was not different between the groups (−1.35 ± 0.87 h for melatonin and −1.51 ± 1.07 h for the placebo group; *P* > 0.05).

**Fig 2:**
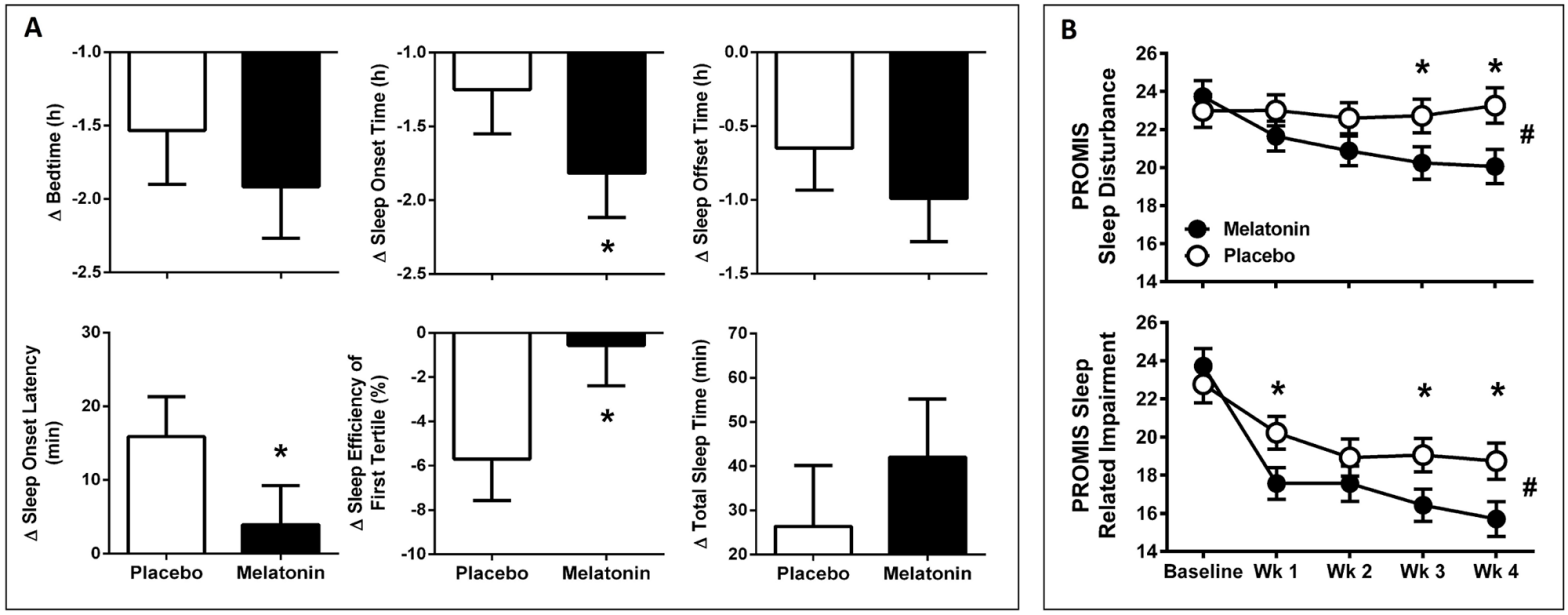
Change in actigraphic sleep parameters and subjective sleep quality and sleep-related impairment under treatment with melatonin and placebo. (A) Predicted change (mean ± 95% CIs) from baseline to treatment phase in actigraphic sleep parameters for individuals during treatment with placebo (open) and 0.5 mg melatonin (closed) (intention-to-treat analyses). Predicted values are derived from regression analyses to account for intra- and inter-individual variability. *P < 0.05. (B) Subjective measures of PROMIS Sleep Disturbance (upper) and PROMIS Sleep Related Impairment (lower) (*n =* 116) at baseline and weeks 1–4 treatment with placebo (open symbols) and melatonin (closed symbols). Data are mean ± SE. *P < 0.05 for post hoc between groups *t* test; #*P* < 0.05 for treatment group x time. CI, confidence interval; PROMIS, Patient Reported Outcomes Measurement Information System; SE, standard error.

Earlier bedtime in the placebo condition was associated with longer SOL and lower SE, which was improved with melatonin treatment (Table 2, Fig 2). Relative to baseline, compared to placebo, the melatonin group had 18.2-min-shorter subjective (95% confidence interval [CI] −30.78 to −5.59; *P* = 0.005) and 11.9-min-shorter actigraphic (95% CI −19.54 to −4.39; *P* = 0.002) SOL, 44-min-earlier subjective (95% CI −66 to −21; *P* < 0.001) and 34-min-earlier actigraphic (95% CI −60 to −8; *P =* 0.011) sleep onset time, 38-min-earlier subjective sleep offset time (95% CI −63 to −13; *P* = 0.003), 3.0% higher SE for the entire sleep episode (95% CI 1.02–4.99; *P* = 0.003), and 5.14% higher SE T1 (95% CI 2.52–7.76; *P* < 0.001) (Table 2, Fig 2a).

**Table 2:** Subjective and objective sleep parameters at baseline and during nights of treatment with trial medication.

	Baseline		All treatment nights		Treatment versus baseline by group				Difference between groups’ change from baseline to treatment	
	Placebo	Melatonin	Placebo	Melatonin	Placebo		Melatonin		β (95% CI)	*P*
					Diff (95% CI)	*P*	Diff (95% CI)	*P*		
**Actigraphy**										
No.: participants; nights	55; 373	56; 378	49; 1,029	54; 1,125						
No. nights per patient: Mean ± SD	6.78 ± 0.53	6.75 ± 0.55	21.00 ± 3.63	20.83 ± 4.24					0.13 (−1.67 to 1.40)	0.862
Bedtime (hh:mm): Mean ± SD	00:58 ± 01:49	01:11 ± 1:52	23:23 ± 1:30	23:15 ± 1:23	**−1:35 (−1:58 to −1:13)**	**<0.001**	**−1:57 (−2:19 to −1:35)**	**<0.001**	−0:23 (−0:54 to 0:08)	0.142
Sleep onset time (hh:mm)^a^: Mean ± SD	01:20 ± 01:49	01:32 ± 1:54	**23:59 ± 1:37**	**23:41 ± 1:35** ^b^	**−1:17 (−1:35 to −0:58)**	**<0.001**	**−1:52 (−2:11 to −1:34)**	**<0.001**	**−0:34 (−1:00 to −0:08)**	**0**.**011**
Sleep offset time (hh:mm): Mean ± SD	08:58 ± 1:57	08:56 ± 2:07	08:11 ± 1:50	07:53 ± 1:43	**−0:43 (−1:01 to −0:24)**	**<0.001**	**−1:01 (−1:19 to −0:43)**	**<0.001**	−0:21 (−0:46 to 0:04)	0.095
Rise time (hh:mm): Mean ± SD	09:13 ± 1:56	09:09 ± 2:06	08:30 ± 1:47	08:09 ± 1:39	**−0:36 (−0:54 to −0:17)**	**<0.001**	**−0:57 (−1:14 to −0:40)**	**<0.001**	−0:24 (−0:48 to 0:00)	0.051
TIB (h): Mean ± SD	8.24 ± 1.75	7.98 ± 1.81	9.10 ± 1.51	8.90 ± 1.54	**0.96 (0.67–1.24)**	**<0.001**	**0.98 (0.70–1.26)**	**<0.001**	−0.01 (−0.40 to 0.37)	0.949
TST (h): Mean ± SD	6.80 ± 1.51	6.62 ± 1.65	7.20 ± 1.44	7.34 ± 1.37	**0.41 (0.17–0.65)**	**0.001**	**0.74 (0.51–0.97)**	**<0.001**	0.26 (−0.06 to 0.58)	0.111
SOL (min): Mean ± SD	20.71 ± 26.21	21.14 ± 27.33	**35.58 ± 38.99**	**25.37 ± 27.90**^b^	**17.45 (11.74–23.15)**	**<0.001**	3.67 (−1.90 to 9.23)	0.197	**−11.93 (−19.54 to −4.39)**	**0**.**002**
Median (IQR)	14.0 (2.5–27.0)	12.0 (3.0–29.0)	24.0 (9.0–48.0)	18.0 (6.0–35.0)						
SE (%): Mean ± SD	82.78 ± 7.17	83.13 ± 8.21	**79.22 ± 10.14**	**82.67 ± 7.97**^b^	**−3.48 (−4.93 to −2.03)**	**<0.001**	−0.06 (−1.47 to 1.35)	0.935	**3.01 (1.02–4.99)**	**0**.**003**
Median (IQR)	83.7 (78.2–88.0)	84.3 (79.0–89.1)	81.0 (74.6–86.0)	84.2 (79.0–88.3)						
SE tertile 1 (%)^a^: Mean ± SD	83.78 ± 9.50	83.19 ± 12.47	**78.37 ± 12.81**	**82.49 ± 10.45** ^b^	**−6.13 (−8.14 to −4.13)**	**<0.001**	−0.68 (−2.63 to 1.27)	0.493	**5.14 (2.52–7.76)**	**<0**.**001**
Median (IQR)	85.0 (78.9–90.7)	86.2 (77.7–91.7)	80.8 (73.5–87.4)	84.6 (77.9–89.9)						
SE tertile 2 (%): Mean ± SD	89.57 ± 5.92	90.11 ± 6.15	88.09 ± 8.01	89.72 ± 8.05	**−1.32 (−2.38 to −0.27)**	**0.014**	−0.62 (−1.64 to 0.39)	0.229	0.96 (−0.43 to 2.35)	0.177
Median (IQR)	90.5 (86.8–93.6)	91.3 (87.8–94.2)	89.8 (86.0–92.8)	91.7 (87.9–94.3)						
SE tertile 3 (%): Mean ± SD	85.03 ± 8.30	85.98 ± 7.67	84.49 ± 8.44	86.16 ± 7.59	−0.36 (−1.38 to 0.67)	0.490	−0.20 (−1.19 to 0.79)	0.690	0.37 (−1.07 to 1.81)	0.616
Median (IQR)	86.8 (80.6–90.7)	87.5 (82.6–91.5)	86.1 (80.8–90.2)	87.8 (82.7–91.4)						
WASO (min): Mean ± SD	49.81 ± 25.84	45.44 ± 26.38	59.17 ± 31.29	52.04 ± 31.29	**9.34 (4.57–14.12)**	**<0.001**	**7.61 (2.97–12.24)**	**0.001**	−3.09 (−9.32 to 3.14)	0.331
Median (IQR)	47.0 (31.0–63.0)	39.0 (27.0–56.0)	53.0 (39.0–70.5)	44.0 (32.0–62.0)						
**Sleep Diary**										
No.: participants; nights	58; 394	58; 395	49; 1029	54; 1125						
No. nights: Mean ± SD	6.79 ± 0.49	6.81 ± 0.51	21.00 ± 3.63	20.83 ± 4.24					−0.18 (−1.70 to 1.33)	0.811
Bedtime (hh:mm): Mean ± SD	00:46 ± 1:44	01:01 ± 1:43	23:06 ± 1:18	23:01 ± 1:20	**−1:41 (−1:59 to −1:23)**	**<0.001**	**−2:04 (−2:20 to −1:47)**	**<0.001**	−0:24 (−0:48 to 0:01)	0.056
Sleep onset time (hh:mm): Mean ± SD	01:28 1:43	01:42 ± 1:44	**00:06 ± 1:26**	**23:44 ± 1:30** ^b^	**−1:23 (−1:39 to −1:06)**	**<0.001**	**−2:02 (−2:19 to −1:46)**	**<0.001**	**−0:44 (−1:06 to −0:21)**	**<0**.**001**
Sleep offset time (hh:mm): Mean ± SD	09:01 ± 1:57	09:09 ± 2:06	**08:27 ± 1:45**	**08:00 ± 1:47** ^b^	**−0:34 (−0:53 to −0:16)**	**<0.001**	**−1:05 (−1:23 to −0:48)**	**<0.001**	**−0:38 (−1:03 to −0:13)**	**0**.**003**
ΤΙΒ (h): Mean ± SD	8.25 ± 1.84	8.13 ± 1.76	9.33 ± 1.57	8.93 ± 1.87	**1.10 (0.80–1.40)**	**<0.001**	**0.88 (0.59–1.17)**	**<0.001**	−0.28 (−0.69 to 0.13)	0.179
ΤSΤ (h): Mean ± SD	7.22 ± 1.87	7.08 ± 1.91	7.92 ± 1.68	7.88 ± 2.01	**0.77 (0.45–1.09)**	**<0.001**	**0.94 (0.63–1.24)**	**<0.001**	0.13 (−0.30 to 0.55)	0.563
SOL (min): Mean ± SD	42.32 ± 45.98	41.47 ± 46.40	**60**.**93 ± 60**.**10**	**42**.**72 ± 56**.**39** ^b^	**19.34 (9.40–29.28)**	**<0.001**	1.55 (−8.03 to 11.13)	0.751	**−18**.**18 (−30**.**78 to −5**.**59)**	**0**.**005**
Median (IQR)	30 (15–60)	30 (15–50)	40 (20–90)	30 (15–55)						
SE (%): Mean ± SD	87.41 ± 11.32	87.21 ± 14.17	84.96 ± 13.27	87.65 ± 15.28	−1.68 (−4.31–0.95)	0.211	1.18 (−1.38 to 3.73)	0.366	3.16 (−0.41 to 6.73)	0.083
Median (IQR)	90.8 (82.7–95.7)	92.3 (83.3–96.0)	88.9 (79.8–94.2)	92.3 (85.5–96.2)						
WASO (min): Mean ± SD	19.60 ± 28.14	21.51 ± 39.40	25.74 ± 43.78	21.59 ± 39.93	4.76 (−2.54 to 12.06)	0.201	0.15 (−6.92 to 7.21)	0.968	−5.88 (−15.53 to 3.77)	0.232
Median (IQR)	10 (0–30)	5 (0–30)	10 (0–30)	5 (0–30)						

Median (IQR) presented for variables with skewed distribution.

^a^Underlined variable indicates primary outcome.

^b^Bolded results indicate statistically significant parameters.

Abbreviations: CI, confidence interval; IQR, interquartile range; SD, standard deviation; SE, sleep efficiency; SOL, sleep onset latency; TIB, time in bed; TST, total sleep time; WASO, wake after sleep onset.

**Fig 3:**
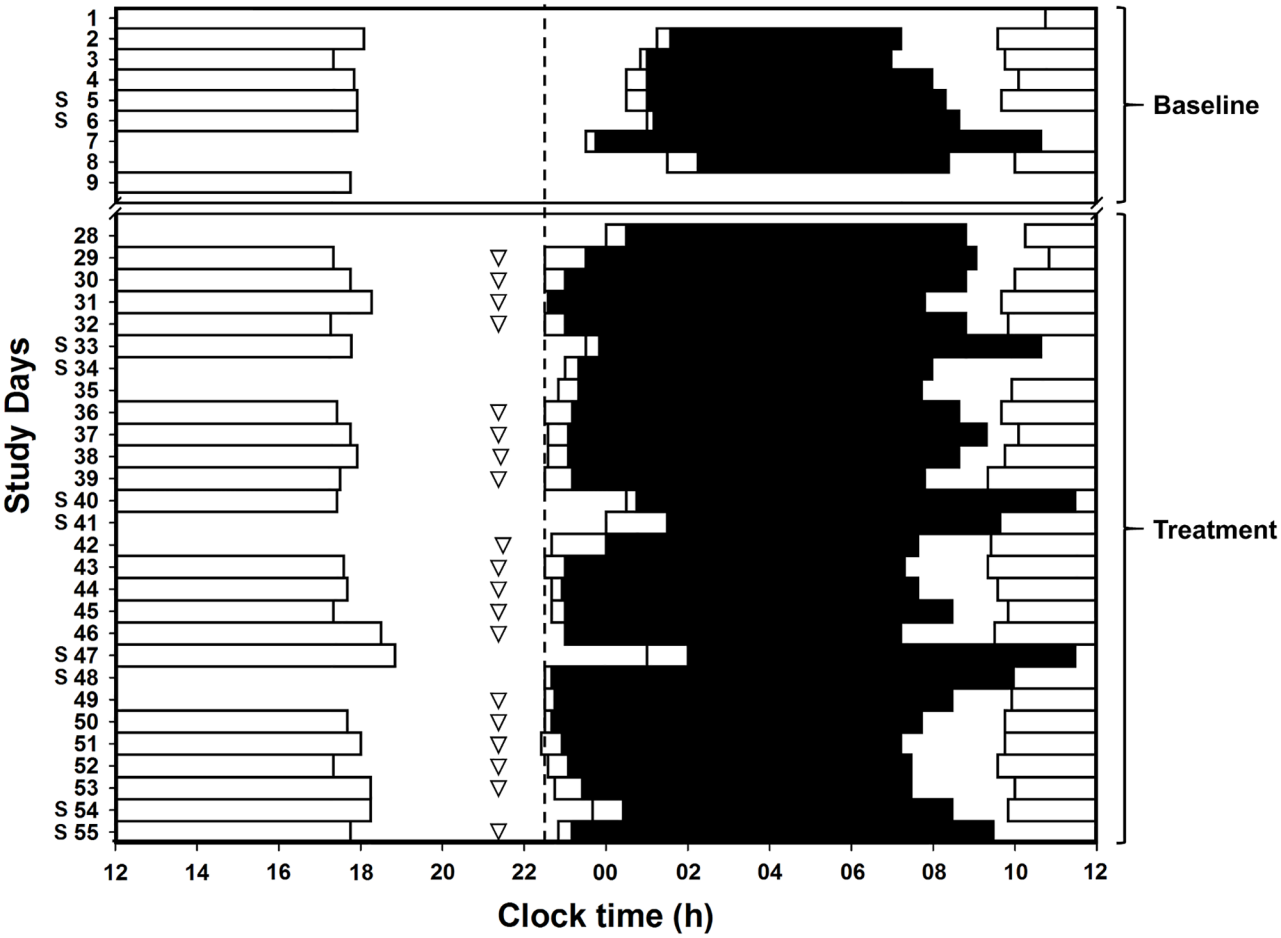
Raster plot of sleep-wake and work patterns for a representative DSWPD patient at baseline and during treatment with 0.5 mg melatonin. Black bars: sleep with open bar illustrating sleep onset time; triangles: time of melatonin administration; dashed line: DBT; open bars: work. On nights prior to daytime commitments on at least 5 nights per week, medication was taken 1 h before DBT identified prior to the study. DBT, desired bedtime; DSWPD, Delayed Sleep-Wake Phase Disorder.

We conducted post hoc analyses of all nights of the treatment period (28 d), including the nights on which treatment was not taken (Table 3). The melatonin group compared to the placebo group had a 29-min-earlier actigraphic sleep onset time (95% CI −54 to −4; *P =* 0.023) and 4.37% greater SE T1 (95% CI 1.91, 6.83; *P =* 0.001). An example of the treatment effect for a participant administered melatonin is plotted in Fig 3.

**Table 3:** Subjective and objective sleep parameters at baseline and during all nights of the 28-d treatment period.

	Baseline		All nights		Difference between treatment groups’ change from baseline to treatment	
	Placebo	Melatonin	Placebo	Melatonin	β (95% CI)	*P*
**Actigraphy**						
No.: participants; nights	55; 373	56; 378	51; 1,404	55; 1,518		
No. nights per patient	6.78 ± 0.53	6.75 ± 0.55	27.53 ± 3.36	27.60 ± 2.97	0.10 (−1.13 to 1.33)	0.869
Bedtime (hh:mm): Mean ± SD	00:58 ± 01:49	01:11 ± 1:52	23:42 ± 1:41	23:38 ± 1:36	−0:20 (−0:50 to 0:11)	0.210
Sleep onset time (hh:mm)^a^: Mean ± SD	01:20 ± 01:49	01:32 ± 1:54	**00:17 ± 1:44**	**00:03 ± 1:37**^b^	**−0:29 (−0:54 to −0:04)**	**0.023**
Sleep offset time (hh:mm): Mean ± SD	08:58 ± 1:57	08:56 ± 2:07	08:25 ± 1:51	08:08 ± 1:50	−0:17 (−0:40, 0:06)	0.144
Rise time (hh:mm): Mean ± SD	09:13 ± 1:56	09:09 ± 2:06	08:43 ± 1:49	08:25 ± 1:47	−0:18 (−0:41, 0:05)	0.111
TIB (h): Mean ± SD	8.24 ± 1.75	7.98 ± 1.81	9.00 ± 1.56	8.77 ± 1.55	0.00 (−0.36, 0.36)	0.995
TST (h): Mean ± SD	6.80 ± 1.51	6.62 ± 1.65	7.17 ± 1.45	7.24 ± 1.39	0.22 (−0.08, 0.52)	0.157
SOL (min): Mean ± SD	20.71 ± 26.21	21.14 ± 27.33	**33.47 ± 37.05**	**24.90 ± 30.51**^b^	**−9.68 (−16.69 to −2.67)**	**0.007**
Median (IQR)	14 (2–27)	12 (3–29)	23 (8–46)	17 (6–34)		
SE (%): Mean ± SD	82.78 ± 7.17	83.13 ± 8.21	**79.73 ± 9.62**	**82.70 ± 8.43**^b^	**2.72 (0.59–4.86)**	**0.013**
Median (IQR)	83.74 (78.26–88.06)	84.30 (79.07–89.22)	81.44 (75.63–86.09)	84.31 (78.97–88.54)		
SE tertile 1 (%)^a^: Mean ± SD	83.78 ± 9.50	83.19 ± 12.47	**79.32 ± 12.35**	**82.87 ± 10.36**^b^	**4.37 (1.91–6.83)**	**0.001**
Median (IQR)	85.26 (78.95–90.65)	86.25 (77.74–91.79)	81.97 (74.29–87.73)	85.01 (78.14–90.27)		
SE tertile 2 (%): Mean ± SD	89.57 ± 5.92	90.11 ± 6.15	88.19 ± 7.76	89.84 ± 8.11	0.98 (−0.33 to 2.30)	0.144
Median (IQR)	90.45 (86.82–93.57)	91,21 (87.74–94.17)	89.98 (85.96–92.75)	91.78 (87.85–94.48)		
SE tertile 3 (%): Mean ± SD	85.03 ± 8.30	85.98 ± 7.67	84.47 ± 8.39	85.79 ± 8.50	0.11 (−1.37 to 1.60)	0.883
Median (IQR)	86.84 (80.56–90.71)	87.57 (82.62–91.48)	85.96 (80.59–90.30)	87.50 (82.17–91.55)		
WASO (min): Mean ± SD	49.81 ± 25.84	45.44 ± 26.38	57.82 ± 29.96	50.31 ± 30.13	−3.13 (−8.88 to 2.61)	0.285
Median (IQR)	47 (31–63)	39.5 (27–56)	52 (38–70)	43 (31–61)		
**Sleep Diary**						
*N*: participants; nights	58; 394	58; 395	51; 1,404	55; 1,518		
No. nights: Mean ± SD	6.79 ± 0.49	6.81 ± 0.51	27.53 ± 3.36	27.60 ± 2.97	0.05 (−1.18 to 1.28)	0.936
Bedtime (hh:mm): Mean ± SD	00:46 ± 1:44	01:01 ± 1:43	23:28± 1:41	23:25 ± 1:37	−0:20 (−0:44 to 0:04)	0.100
Sleep onset time (hh:mm): Mean ± SD	01:28 1:43	01:42 ± 1:44	**00:20± 1:41**	**00:03 ± 1:40**^b^	**−0:34 (−0:56 to −0:12)**	**0.002**
Sleep offset time (hh:mm): Mean ± SD	09:01 ± 1:57	09:09 ± 2:06	**08:41 ± 1:50**	**08:17 ± 1:51**^b^	**−0:35 (−1:00 to −0:11)**	**0.005**
ΤΙΒ (h): Mean ± SD	8.25 ± 1.84	8.13 ± 1.76	9.21 ± 1.72	8.84 ± 1.84	−0.28 (−0.67 to 0.11)	0.164
ΤSΤ (h): Mean ± SD	7.22 ± 1.87	7.08 ± 1.91	7.93 ± 1.79	7.89 ± 1.96	0.08 (−0.33 to 0.49)	0.708
SOL (min): Mean ± SD	42.32 ± 45.98	41.47 ± 46.40	**54.62 ± 56.76**	**38.68 ± 51.09**^b^	**−15.45 (−26.22 to −4.68)**	**0.005**
Median (IQR)	30 (15–60)	30 (15–50)	30 (20–65)	25 (15–45)		
SE (%): Mean ± SD	87.41 ± 11.32	87.21 ± 14.17	86.16 ± 12.84	88.69 ± 14.11	2.78 (−0.64 to 6.21)	0.111
Median (IQR)	90.82 (82.73–95.65)	92.31 (83.33–95.97)	90.20 (81.58–94.85)	93.40 (86.21–96.43)		
WASO (min): Mean ± SD	19.60 ± 28.14	21.51 ± 39.40	23.46 ± 40.80	19.60 ± 36.85	−5.28 (−14.42 to 3.85)	0.257
Median (IQR)	10 (0–30)	5 (0–30)	10 (0–30)	5 (0–20)		

Median (IQR) presented for variables with skewed distribution.

^a^Underlined variable indicates primary outcome.

^b^Bolded results indicate statistically significant parameters.

Abbreviations: CI, confidence interval; IQR, interquartile range; SD, standard deviation; SE, sleep efficiency; SOL, sleep onset latency; TIB, time in bed; TST, total sleep time; WASO, wake after sleep onset.

Sensitivity analyses were undertaken including only those nights on which patients took treatment and went to bed according to the study protocol. Compared to placebo, the melatonin group had earlier actigraphic sleep onset time (26 min; 95% CI −51 to −2; *P =* 0.038), higher SE T1 (5.50%; 95% CI 2.41–8.58; *P* < 0.001), shorter subjective (18.4 min; 95% CI −34.21 to −2.66; *P =* 0.022) and objective (15.7 min; 95% CI −24.41 to −6.89; *P* < 0.001) SOL, earlier subjective sleep onset time (40 min; 95% CI −67 to −14; *P =* 0.004), earlier subjective sleep offset time (42 min; 95% CI −74 to −9; *P =* 0.013), and higher actigraphic SE for the entire sleep episode (3.27%; 95% CI 1.30–5.24, *P =* 0.001).

For PROMIS Sleep Disturbance, a treatment by time interaction effect was found (F_4,408_ = 3.83, *P =* 0.005), and a significant main effect of time (F_4,408_ = 4.01, *P =* 0.003) but no main effect of treatment (*P =* 0.112). Post hoc analysis showed that PROMIS Sleep Disturbance score was lower in the melatonin group at week 3 (*P =* 0.042) and week 4 (*P* = 0.016) compared to placebo (Fig 2b upper). PROMIS Sleep Related Impairment showed a treatment by time interaction effect (F_4,408_ = 4.29, *P =* 0.002), a main effect of time (F_4,408_ = 37.12, *P* < 0.0001), but no main effect of treatment (*P =* 0.102). Melatonin treatment resulted in significantly lower PROMIS score for sleep-related impairment at week 1 (*P =* 0.035), week 3 (*P =* 0.042), and week 4 (*P =* 0.018; Fig 2b lower).

Prior to treatment, participants in the treatment groups were not different on ratings on the ISI, PSQI, ESS, SDS, absenteeism, or presenteeism (Table 4). Compared to placebo, ISI and PSQI were significantly improved with melatonin treatment. Following treatment, the melatonin group reported a lower ISI score (*P =* 0.035) and included more individuals reporting absent insomnia symptoms on the ISI (*P =* 0.008). Mean PSQI score post treatment was lower in the melatonin group compared to placebo (*P =* 0.037). Significant treatment by time interactions were observed for ISI (*P =* 0.004), PSQI (*P =* 0.023), and SDS (*P =* 0.045) (Table 4), with melatonin treatment associated with larger reductions in each score. Absenteeism and presenteeism post treatment were not different between groups.

**Table 4:** Improvements in standardised measures of subjective sleep quality, sleep-related impairment and daytime dysfunction at baseline and post-treatment for the melatonin and placebo treatment groups.

	Baseline			Post-treatment				Post-treatment versus baseline by group		Post-treatment versus baseline between groups *P*
	Placebo (*n =* 58)	Melatonin^a^ (*n =* 58)	Melatonin versus placebo *P*	Placebo (*n =* 47)	Melatonin (*n =* 48)	Melatonin versus placebo		Placebo	Melatonin	
						Difference (95% CI)^c^	*P*	Difference (95% CI)^c^	Difference (95% CI)^c^	
ISI (mean ± SD)	12.29 ± 4.67	12.93 ± 4.86	0.483	10.70 ± 5.2	8.33 ± 5.57	−2.37 (−4.56 to −0.18)	**0**.**035**	−1.59 (−3.10 to −0.08)	−4.59 (−5.96 to −3.22)	**0**.**004**
Categorical ISI: *n* (%)						0.28 (0.12–0.63)	**0**.**008**	0.55 (0.32–0.96)	0.14 (0.07–0.26)	**0**.**001**
Absent (0–7)	8 (13.8)	9 (16.4)	0.713	13 (27.7)	27 (56.3)					
Subthreshold (8–14)	30 (51.7)	24 (43.6)		22 (46.8)	17 (35.4)					
Moderate (15–21) / Severe (22–28)	20 (34.5)	22 (40.0)		12 (25.5)	4 (8.4)					
PSQI (mean ± SD)	8.41 ± 3.38	8.50 ± 3.20	0.889	7.81 ± 3.35	6.40 ± 3.17	−1.41 (−2.74 to −0.08)	**0**.**037**	−0.61 (−1.51 to 0.30)	−2.10 (−3.03 to −1.18)	**0**.**023**
Categorical PSQI: *n* (%)						0.60 (0.26–1.40)	0.292	0.56 (0.29–1.07)	0.28 (0.14–0.56)	0.158
Normal (0–5)	12 (20.7)	10 (17.9)	0.814	15 (31.9)	21 (43.8)					
Poor Sleep (>5)	46 (79.3)	46 (82.1)		32 (68.1)	27 (56.3)					
ESS (mean ± SD)	5.50 ± 3.59	6.12 ± 3.48	0.337	5.19 ± 3.09	4.88 ± 2.80	−0.32 (−1.52 to 0.89)	0.603	−0.31 (−1.10 to 0.49)	−1.25 (−2.10 to −0.39)	0.115
Categorical ESS: *n* (%)						0.98 (0.19–5.15)	>0.999	0.37 (0.14–0.97)	0.36 (0.10–1.35)	0.978
Normal/borderline (0–9)	49 (84.5)	49 (84.5)	>0.999	44 (93.6)	45 (93.8)					
Excessive daytime sleepiness (>9)	9 (15.5)	9 (15.5)		3 (6.4)	3 (6.3)					
SDS (mean ± SD)	14.79 ± 4.88	14.94 ± 5.13	0.878	12.38 ± 5.64	10.35 ± 6.14	−2.03 (−4.42 to 0.37)	0.097	−2.41 (−3.69 to −1.13)	−4.58 (−6.27 to −2.89)	**0**.**045**
Absenteeism (mean ± SD)	3.45 ± 3.97	2.56 ± 3.85	0.260	2.17 ± 3.31	1.23 ± 2.57	−0.94 (−2.15 to 0.27)	0.126	−1.28 (−2.31 to −0.25)	−1.33 (−2.54 to −0.13)	0.944
Presenteeism (mean ± SD)	10.55 ± 7.44	8.49 ± 6.85	0.174	6.34 ± 6.52	4.54 ± 5.88	−1.80 (−4.33 to 0.74)	0.162	−4.21 (−6.54 to −1.88)	−3.95 (−6.13 to −1.77)	0.871
PGI-C, *n* (%)^b^						1.65 (0.76–3.62)	0.239			
No change to a little better (1–3)				27 (55.1)	23 (42.6)					
Somewhat better to a great deal better (4–7)				22 (44.9)	31 (57.4)					

^a^For the melatonin group at baseline; ISI *n =* 51; PSQI *n =* 52; SDS *n =* 52; absenteeism *n =* 51; presenteeism *n =* 51.

^b^For PGI-C at post-treatment: placebo *n =* 49; melatonin *n =* 54.

^c^Differences presented for categorical analyses for ISI, PSQI, ESS, and PGI-C are odds ratios.

Abbreviations: ESS, Epworth Sleepiness Scale; ISI, Insomnia Severity Index; PGI-C, Patients’ Global Impression of Change Scale; PSQI, Pittsburgh Sleep Quality Index; SDS, Sheehan Disability Scale.

CGI Global Improvement ratings were significantly different between treatment groups (*P =* 0.011), with 52.8% (*n =* 28) of the melatonin group classified as very much/much improved compared to 24.0% (*n =* 12) of the placebo group (Table 5). CGI Efficacy Index was lower following melatonin treatment compared to placebo (*P =* 0.045). The proportion of patients in the CGI Severity categories did not change following treatment (Table 5). Post-treatment PGI-C scores did not differ between groups.

**Table 5:** Clinician-rated measures of sleep-related impairment from the CGI scale for treatment groups at baseline and post treatment.

	Baseline		Post-treatment		*P*^a^
	Placebo (*n =* 58)	Melatonin (*n =* 58)	Placebo (*n =* 50)	Melatonin (*n =* 54)	
**Severity, no. (%)**					
Normal to mildly ill (1–3)	19 (38.0)	19 (35.3)	25 (50.0)	34 (62.2)	0.365
Moderately ill (4)	22 (44.0)	29 (53.7)	22 (44.0)	17 (31.5)	
Markedly ill to amongst the most extreme (5–7)	9 (18.0)	6 (11.2)	3 (6.0)	3 (5.6)	
**Global improvement, no. (%)**^b^					
Very much to much improved (1–2)			12 (24.0)	28 (52.8)	**0**.**011**
Minimally improved to minimally worse (3–5)			37 (74.0)	24 (45.3)	
Much worse to very much worse (6–7)			1 (2.0)	1 (1.9)	
**Efficacy index**			8.60 ± 4.32	6.87 ± 4.35	**0**.**045**

^a^Chi-squared comparison between groups post treatment.

^b^Global Improvement melatonin group *n =* 53.

Abbreviation: CGI, Clinical Global Impression.

Post-treatment DLMO could not be calculated for 6 of the subset of 49 participants; melatonin concentrations were above threshold (2.3 pg/ml) at the start of the collection phase in 4 participants (*n =* 3 melatonin, *n =* 1 placebo), with higher levels in 1 participant indicating that melatonin treatment was taken on the night of assessment. Concentrations did not cross threshold for 2 participants (*n =* 1 melatonin, *n =* 1 placebo). Following melatonin treatment (*n =* 23), DLMO was advanced by 0.73 ± 1.21 h, compared to 0.24 ± 1.11 h following placebo (*n =* 20) although this difference did not reach statistical significance (*P* > 0.05).

No deaths or serious adverse events were reported for either treatment group, and no participants discontinued due to adverse events. Adverse event rates did not differ between the treatment groups (Table 6). Adverse events did not occur in more than 5% of patients taking melatonin. Unusual dreaming was reported by 7% of the placebo group compared to 2% of the melatonin group.

**Table 6:** Categorisation of adverse events reported in the placebo and melatonin treatment groups.

Category of symptoms	Report frequency for each treatment group
Light-headedness	*n =* 2 melatonin
	*n =* 2 placebo
Daytime sleepiness	*n =* 2 melatonin
	*n =* 1 placebo
Decreased libido	*n =* 2 melatonin
Headache	*n =* 1 melatonin
Vivid dreams and/or nightmares	*n =* 1 melatonin
	*n =* 4 placebo

In post hoc analysis, we assessed whether the degree of ‘social jetlag’ observed in our patients was affected by our melatonin treatment protocol. Social jetlag has been described as the discrepancy between sleep timing on work and free days, thought to reflect the discrepancy between social and biological time [39]. We operationalised this definition in our protocol by comparing sleep timing on nights when medication was taken compared to when medication was not taken because this was thought to reflect work and free nights, respectively. Data were included when at least 2 nonmedication nights were available for each participant (*n =* 83). Consistent with previous studies, sleep timing was defined by mid-sleep, calculated here as the midpoint between actigraphic sleep onset and sleep offset time. Median social jetlag was not different between the melatonin group (1.17 [0.70–1.75], median [IQR]) and the placebo group (0.89 [0.58–1.62]) (*P* > 0.05).

## Discussion

In a sample of DSWPD patients with confirmed circadian misalignment, compared to placebo, 0.5 mg melatonin taken 1 h prior to patient-DBT in a pragmatic treatment protocol resulted in earlier timing of sleep onset, improved sleep quality, and reduced sleep-related impairments. Melatonin treatment reduced patient-reported sleep disturbance, insomnia severity, and daytime functional disability and was associated with larger clinician-rated improvements in symptoms. This is the largest placebo-controlled trial to assess the clinical efficacy of melatonin to improve sleep initiation and sleep quality in DSWPD. The trial has assessed the effects of melatonin when sleep is attempted at the patient-DBT in DSWPD patients with confirmed circadian misalignment, using patient-reported (PROMIS, PSQI, ISI, SDS, PGI-C), clinician-assessed (CGI-Global Improvement and Efficacy), and objective (actigraphic sleep onset time, SOL, SE) assessments of sleep quality and daytime function.

We instructed patients to attempt sleep at their DBT during treatment, which resulted in the sleep episode starting, on average, 2 h earlier than habitual bedtime during baseline. The earlier timing in bedtime resulted in longer SOL for those on placebo, as expected for individuals attempting to sleep earlier in their circadian cycle [40]. Treatment with melatonin reduced the time taken to fall asleep to near baseline levels and resulted in a 34-min-earlier sleep onset and 36-min-earlier self-reported sleep offset time compared to placebo, exceeding consensus recommendations of critical clinical significance thresholds for improvements in these outcomes in DSWPD (15 min in both cases) [15]. Actigraphically recorded SE during the first tertile of sleep and overall SE were also improved with melatonin treatment.

Melatonin treatment produced considerable improvements in subjective sleep complaints. Patients taking melatonin reported improved sleep quality and reduced severity of insomnia symptoms. A higher proportion of those taking melatonin also reported an absence of insomnia symptoms. To assess the clinical utility of melatonin for DSWPD, the observed effect sizes should be compared to other treatments for DSWPD, ideally. Unfortunately, to our knowledge, such studies are lacking. The effects reported for subjective SOL are comparable to those of first-line therapies for insomnia, including cognitive behavioural therapy for insomnia [41] and Z-class hypnotic agents [42]. The reduction in subjective insomnia severity is greater than reported with Suvorexant for treatment of primary insomnia [43]. Our findings of reduced functional impairments in work/school, social, and family life extend previous reports of improvements in school performance in adolescents [44] and quality of life in adults [45] following melatonin treatment. After only 1 wk of treatment with melatonin, patient-reported sleep-related impairment was significantly improved relative to placebo. The 4-point reduction in PROMIS sleep-related impairment is comparable to the 5-point larger reduction reported for cognitive behavioural therapy for insomnia modified for bipolar disorder compared to psychoeducation [46] as well as the 4-point difference reported between individuals with a recent mild traumatic brain injury and matched controls [47]. We suggest that these improvements in daytime functional measures were secondary to the observed improvements in sleep quality.

Melatonin treatment advanced the timing of DLMO by 36 min more than with placebo (0.73 versus 0.24 h), although this difference was not significant. There are several possible reasons for this lack of difference in circadian phase shifting. Melatonin was scheduled to be taken 1 h prior to DBT to facilitate sleep onset primarily through the direct, sleep-promoting effects of the hormone [16] during the wake maintenance zone, when sleep propensity is low [2,48]. In contrast, most previous trials of melatonin for DSWPD have focussed on the circadian phase shifting properties of melatonin, which require that treatment be administered several hours before the timing of habitual sleep or endogenous melatonin onset [13,14]. Although melatonin was administered on average 1.4 h before DLMO—which may be expected to induce a modest phase advance [33,49]—because assessment of circadian phase shifting was performed in only a subset of patients, the study may have been underpowered to detect significant differences. Second, by instructing patients to attempt sleep at their DBT (which may be the required bedtime for some individuals), the light-dark cycle was phase advanced, resulting in an advance in the timing of the circadian pacemaker in both groups [50]. Finally, our pragmatic treatment protocol required patients to take melatonin and sleep at their DBT—which is typically earlier than the habitual bedtime—on at least 5 nights per week, to accommodate work or school activities. On nights when patients did not sleep at their DBT or take melatonin, the circadian pacemaker may have reverted to a delayed pattern, thus counteracting the previous melatonin-induced phase advances. As an indirect consequence of a protocol that allows patients to choose their own sleep-wake schedule, social jetlag (i.e., the difference in timing between treatment and nontreatment nights) could be increased, which may have health implications [51]. We did not find a significant difference in the degree of social jetlag between the melatonin and placebo groups.

Current melatonin treatment protocols for DSWPD vary in patient diagnostic and selection criteria as well as in the dose, formulation, and timing of melatonin, and they may result in low compliance, thus limiting translation into clinical practice. We improved on previous academic-led trials by selecting patients who have an endogenous circadian basis to their symptoms [52]. Furthermore, we have developed a pragmatic treatment protocol that allows patients to improve sleep initiation when needed to accommodate daytime commitments the following morning and found a high rate of compliance. This approach of allowing patients to optimise treatment administration to align with clinical and lifestyle needs is highly novel and, to our knowledge, has not been previously reported in the literature. The behavioural intervention of attempting sleep at the earlier DBT facilitated earlier timing of sleep onset, as reflected by the significant improvements in sleep outcomes in the placebo group (Tables 2 and 4). These findings demonstrate that behavioural approaches for managing DSWPD appear to be highly effective, potentially through altered light-dark cues. The improvements in sleep produced by the behavioural intervention were, however, considerably enhanced by melatonin administration. This is important information for clinicians seeking an evidence-based therapeutic approach for DSWPD. We used a lower dose formulation than those typically used previously (3–5 mg) [10–12,14,44,53]. Our results demonstrate that large doses of melatonin are not required for improvements in sleep initiation and daytime functioning in DSWPD patients, noting, however, that a dose-response relationship has previously been reported [36].

Treatment of a circadian disorder with melatonin would be preferable to a traditional hypnotic given that melatonin both facilitates sleep and shifts the timing of the circadian pacemaker [16]. There is a lack of evidence of the value of hypnosedatives in the treatment of phenotyped DSWPD and a large body of literature on the adverse effects of such medications. In addition to the properties of benzodiazepines as a muscle relaxant, the use of this class of drugs is often associated with residual sleepiness the following morning, cognitive impairments including memory, and issues of dependence and withdrawal [54]. The overdose death rate with benzodiazepine use has increased from 0.58 to 3.07 per 100,000 adults [55]. A potentially important clinical application for melatonin is in its use to facilitate discontinuation of benzodiazepines [56].

Limitations of the study should be considered. The original sample size was not reached due to slower-than-anticipated recruitment and financial constraints. Statistical significance was still achieved in the main outcomes, probably because we only included patients with delayed circadian phase relative to DBT, melatonin treatment was timed before DLMO, and we continued treatment for 4 wk, compared to the variable protocols used in the studies that informed our original sample size estimate [11,57–59]. The implications of smaller sample size should be considered, including internal and external validity of the findings. Testing of efficacy and safety were limited to a 4-wk interval. We did not assess clinical benefits or safety of melatonin with long-term treatment, nor did we do a follow-up assessment after treatment was discontinued. Furthermore, it remains unknown whether the same treatment regime would benefit all patients who meet current (ICSD symptom-based) diagnostic criteria for DSWPD but do not undergo further classification based on circadian phase. In our study, patients were diagnosed via clinical interview based on ICSD-2 criteria. An updated version of the ICSD incorporating recommendations to consider objective assessments, including circadian phase, is now available.

Patients experiencing DSWPD symptoms without a circadian delay are not likely to be sleeping in their wake maintenance zone, and therefore the soporific effects of melatonin may be diminished. One-third of patients diagnosed with DSWPD in the current study did not present circadian misalignment between DLMO and DBT. It is important to include circadian phase assessment in the diagnosis and management of DSWPD. It should also be noted that some individuals with delayed sleep due to non-circadian reasons could ultimately experience delayed circadian timing due to delayed light-dark exposure on an ongoing basis. This has implications for the growing prevalence of DSWPD and for treatment protocols such as cognitive behavioural therapy for insomnia, which includes the practice of stimulus control, in which patients can be encouraged to rise from bed in the evening if they are unable to fall asleep, subsequently exposing themselves to phase-delaying light.

Although the formulation of melatonin used in this trial was certified for its purity and quality, access to pharmaceutical-grade melatonin is variable across different countries. In numerous countries, a compounding pharmacist must deliver an accurate dosage of melatonin. In countries such as the United States in which melatonin is not regulated, however, issues of quality, dose, and preparation are likely to be a concern for clinicians. These practical limitations, together with the lack of access to circadian phase assessments, present some barriers to translation of the current findings. This trial does, however, support the clinical utility of circadian diagnostic and treatment approaches in order to bring clinical practice in line with scientific discoveries in the field.

We showed that short-term, low-dose melatonin administration in a pragmatic treatment protocol with behavioural sleep-wake scheduling is an efficacious and safe treatment for DSWPD patients with confirmed circadian misalignment, resulting in improvements in objective and subjective sleep quality, daytime function, and clinical symptom severity.

## Supporting information

S1 CONSORT Checklist (DOC)

S1 Dataset Demographics. (XLSX)

S2 Dataset Subjective clinician. (XLSX)

S3 Dataset Sleep ITT databases. (XLSX)

S4 Dataset Sleep All nights treatment. (XLSX)

S5 Dataset Social jetlag. (XLSX)

## Abbreviations

BDI: Beck Depression Inventory
CGI-S: Clinical Global Impression-Severity
CI: confidence interval
DBT: desired bedtime
DelSoM: Delayed Sleep on Melatonin
DLMO: dim light melatonin onset
DSWPD: Delayed Sleep-Wake Phase Disorder
ESS: Epworth Sleepiness Scale
ISI: Insomnia Severity Index
PGI-C: Patients’ Global Impression of Change
PROMIS: Patient Reported Outcomes Measurement Information System
PSQI: Pittsburgh Sleep Quality Index
SDS: Sheehan Disability Scale
SE: sleep efficiency
SE T1: sleep efficiency in the first tertile of time in bed
SOL: sleep onset latency
TIB: time in bed
TST: total sleep time
WASO: wake after sleep onset
